# Protocatechuic Acid from *Euonymus alatus* Mitigates Scopolamine-Induced Memory Impairment in Mice

**DOI:** 10.3390/foods13172664

**Published:** 2024-08-23

**Authors:** Yoonsu Kim, Minjung Cho, Jeong Soon Lee, Jisun Oh, Jinkyu Lim

**Affiliations:** 1 Department of Integrative Biology, Kyungpook National University, Daegu 41566, Republic of Korea; yunsu531@gmail.com (Y.K.); cho981023@gmail.com (M.C.); 2 Forest Environment Research Institute of Gyeongsangbuk-do, Gyeongju 38174, Republic of Korea; ljs7942@korea.kr; 3 New Drug Development Center, Daegu-Gyeongbuk Medical Innovation Foundation, Daegu 41061, Republic of Korea; 4 School of Food Science and Biotechnology, Kyungpook National University, Daegu 41566, Republic of Korea

**Keywords:** protocatechuic acid, oxidative stress, memory impairment, bioactive compounds, antioxidation, neuroprotection

## Abstract

The increasing prevalence of age-related neurodegenerative disorders owing to the aging population worldwide poses substantial challenges. This study investigated the neuroprotective effects of protocatechuic acid (PCA), a compound found in various fruits, vegetables, and grains, using a scopolamine-induced hypomnesia mouse model. Six-week-old male C57BL/6J mice were orally administered PCA at doses of 10 and 100 mg/kg body weight per day for two weeks, along with intraperitoneal injections of scopolamine. Learning and memory abilities were assessed using the passive avoidance, Morris water maze, and Y-maze behavioral assays. Biochemical analyses evaluated the levels of oxidative stress markers, including 8-hydroxydeoxyguanosine (8-OHdG) in the blood and malondialdehyde (MDA) in the brain, as well as phase II antioxidant proteins in the hippocampus. Histological examination was conducted to determine hippocampal integrity. Our results demonstrated that PCA administration at 10 mg/kg body weight per day or higher for two weeks (i) significantly ameliorated scopolamine-induced learning and memory impairments, as evidenced by improved performance in behavioral tasks, (ii) reduced plasma 8-OHdG levels and cerebral MDA levels in a dose-dependent manner, (iii) increased antioxidant protein expressions in the hippocampal tissue, and (iv) mitigated histological damage in the hippocampal region of the brain. These findings suggest that oral administration of PCA provides neuroprotective effects against oxidative stress-induced learning and memory impairments, possibly through upregulating antioxidant machinery. Therefore, PCA may serve as a promising dietary supplement for mitigating cognitive deficits associated with neurodegenerative diseases.

## 1. Introduction

The global demographic shift toward an aging population has led to increased incidences of age-related cognitive decline and neurodegenerative disorders, including Alzheimer’s and Parkinson’s diseases [1,2,3,4,5]. Our previous study demonstrated that the ethanolic extract from *Euonymus alatus* (EA) leaves exhibits neuroprotective effects by improving learning and memory in mice subjected to scopolamine-induced impairment, likely through the activation of antioxidant defense mechanisms in the hippocampus [6]. Additionally, EA treatment ameliorated Alzheimer’s disease symptoms in 5XFAD transgenic mice by suppressing neuroinflammation [7].

For centuries, EA, an edible material, has traditionally been used for medicinal purposes across various Asian regions. It has been employed to address a wide range of health conditions, including those related to cancer, hyperglycemia, diabetic complications, inflammation, and detoxification [8,9,10,11,12,13]. Multiple studies have reported that *Euonymus* species contain diverse chemical constituents, such as quercetin, kaempferol, caffeic acid, and protocatechuic acid (PCA), which exhibit various biologically beneficial effects, including antioxidant properties [10,14,15,16]. 

PCA, a phenolic acid chemically known as 3,4-dihydroxybenzoic acid, is naturally present in edible medicinal plants as a secondary metabolite. Its pharmacological activity is primarily attributed to its antioxidant and anti-inflammatory properties [17,18,19].

Maintaining a redox balance in a living system is crucial to counteract oxidative stress, a key factor contributing to age-related neurodegeneration and cognitive decline [20,21,22,23,24,25]. The aging brain often exhibits a compromised antioxidant defense system, characterized by reduced activity of antioxidant enzymes, such as superoxide dismutase (SOD), catalase, glutathione peroxidase, and glutathione reductase. NF-E2-related factor 2 (Nrf2) is a transcription factor that plays a vital role in inducing the expressions of detoxifying and antioxidant enzymes after binding to the antioxidant response element, a process that helps safeguard neural cells from oxidative stress-induced cell death and brain dysfunction [25,26,27,28].

Given the significant role of oxidative stress in neurodegeneration, we investigated the neuroprotective potential of PCA based on its ability to maintain redox balance and activate the Nrf2 signaling pathway in a rodent model of oxidative stress-induced cognitive deficits. Scopolamine (SCO), a muscarinic cholinergic receptor antagonist, disrupts oxidative homeostasis in the brain and is commonly used in research to induce oxidative damage and memory impairment in rodent models [29,30,31,32]. Herein, we examined whether PCA could ameliorate learning and memory deficits by activating the Nrf2 signaling pathway following SCO-induced oxidative stress in mice.

## 2. Materials and Methods

### 2.1. Sample Preparation

The test compound, PCA, was either purchased from Sigma-Aldrich (St. Louis, MO, USA) or isolated from the ethanolic extract of EA leaves (provided by the Forest Environment Research Institute of Gyeongsangbuk-do, Gyeongju, Republic of Korea). The EA leaf extract (EAE) was prepared as previously described [6,7]. Briefly, 50 g of dried EA leaves were immersed in 1 L of 80% (*v*/*v*) ethanol and agitated at 150 rpm and 25 °C for 24 h. The resulting supernatant was filtered through filter paper (Hyundai Micro, Seoul, Republic of Korea), vacuum-evaporated, and freeze-dried. The final preparations were stored at −20 °C until use. The extraction yield was approximately 34.2 g/kg dry weight of EA leaf. 

### 2.2. Experimental Design for Animal Study

Six-week-old male C57BL/6J mice (Hyochang Science, Daegu, Republic of Korea) were used in this study. The mice were maintained under a 12 h light/dark cycle, which began at 07:00, at 22 ± 2 °C and a relative humidity of 50 ± 5%, with free access to water and a regular diet (AIN-76A chow, Hyochang Science, Daegu, Republic of Korea). All animal procedures were conducted in accordance with the guidelines of the Committee on the Care and Use of Laboratory Animals of Kyungpook National University (approval number: 2022-0366).

**Figure 1:**
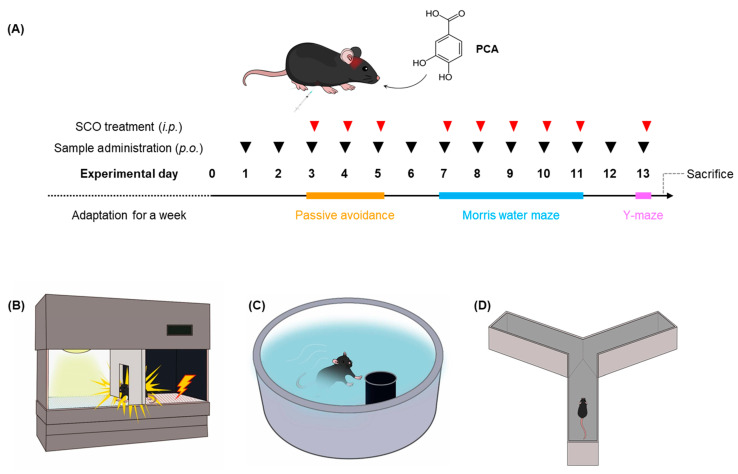
Overview of the animal experiment. (**A**) C57BL/6J mice were allocated into six groups (*n* = 8 per group) and orally administered PCA at 10 and 100 mg/kg BW/day or EAE 150 mg/kg BW/day for 13 days (black triangles). SCO was intraperitoneally injected at 1 mg/kg BW/day 30 min before each session of behavioral tasks (red triangles). (**B**–**D**) Behavioral assessments were conducted according to the schedule using the passive avoidance task (**B**), Morris water maze task (**C**), and Y-maze task (**D**).

A total of 48 mice, weighing an average of 18 ± 1 g, were randomly assigned to 6 groups (8 mice per group) and treated daily as follows (Table 1; Figure 1): (1) control (vehicle only), (2) SCO + vehicle (SCO alone at 1 mg/kg body weight (BW)), (3) SCO and donepezil (DPZ, 5 mg/kg BW), (4) SCO and EAE (150 mg/kg BW), (5) SCO and PCA at a relatively lower dose (PCA_L, 10 mg/kg BW), and (6) SCO and PCA at a relatively higher dose (PCA_H, 100 mg/kg BW). The vehicle consisted of saline containing 2% (*v*/*v*) Tween^®^-80. SCO was intraperitoneally injected, while DPZ (a positive control), EAE, and PCA were administered orally daily for 13 days (Figure 1A). SCO treatment was performed 30 min before each session of behavioral tasks to induce memory impairment in the mice (Figure 1B–D). Following the completion of behavioral testing, the mice were sacrificed and dissected. The brain, liver, and plasma were collected, frozen in liquid nitrogen, and stored at −80 °C for further analyses.

### 2.3. Behavioral Tests

The passive avoidance, Morris water maze, and Y-maze tasks were conducted to evaluate the learning and memory abilities of SCO-treated mice with or without PCA administration, according to the previously described protocols [6,7,33].

The passive avoidance task evaluated aversive learning and memory using the Gemini avoidance system (San Diego, CA, USA). The equipment comprises dark and bright chambers separated by a guillotine door. During the initial test session, mice were introduced to the bright chamber with the door open for 1 min to familiarize themselves with the apparatus. In the subsequent training phase, each mouse was placed in the dark chamber with the door closed. After a 10 s interval, the door was opened, then closed immediately upon the mouse entering the dark chamber, followed by the administration of an electrical foot shock at 0.5 mA for 3 s through the stainless-steel rods in the dark chamber. The following day, during the test phase, each mouse was positioned in the dark chamber with the door shut. After 10 s, the door was opened, and the time the mouse took to move from the dark chamber to the bright chamber was recorded; a prolonged latency period in the dark chamber indicated deficient associative learning and memory, while reduced escape latency exhibited improved memory against SCO insult.

The Morris water maze task evaluated spatial learning and memory in a circular swimming pool with a diameter of 90 cm and a height of 45 cm, featuring a plain inner surface. The pool was filled with water to a depth of 28 cm at a temperature of 22 ± 2 °C. On the first day, mice were allowed to swim freely for 1 min for adaptation. Subsequently, a black platform with a diameter of 6 cm and a height of 29 cm was positioned in one of the quadrants. The water was rendered opaque using non-toxic poster paint. Over the next three days, each mouse underwent three trials per session per day to locate the platform. If a mouse failed to find the platform within 60 s, it was directed to the platform and allowed to remain there for 10 s. On the fifth day, the platform was submerged 1 cm below the opaque water surface in the same quadrant. The latency for each mouse to locate the platform was recorded; a longer latency or failure to reach the platform indicated impaired spatial learning and memory, while a shorter latency indicated improved cognitive performance.

The Y-maze task evaluated working memory by observing spontaneous alternation behavior in a Y-shaped maze. During the test, each mouse was initially placed at the end of one arm of the maze, and their two different arm entries and sequence of entries were meticulously documented for 300 min. Spontaneous alternations were identified as consecutive sets of three different arm choices. The alteration percentage was calculated using the following formula: % alternation = [(number of alternations)/(total arm entries − 2)] × 100.

### 2.4. Histological Analysis 

The brain tissues from three mice per group were dissected, fixed in formalin, embedded in paraffin blocks, and sectioned at a thickness of 5 µm using a microtome (Leica, Nussloch, Germany). The sections were then placed on microscope slides (Thermo Fisher Scientific, Waltham, MA, USA) and stained with hematoxylin and eosin (H&E; Sigma-Aldrich) according to the established protocol [6,7,33]. The stained sections were imaged using a microscope (Eclipse 80i, Nikon, Tokyo, Japan).

### 2.5. Determination of Oxidative Stress Levels

The byproduct levels of oxidative stress-induced lipid peroxidation were assessed by measuring malondialdehyde (MDA) in each tissue homogenate. The cerebral cortex from each dissected brain was homogenized in a lysis buffer (0.1 M phosphate buffer, pH 7.4). After centrifugation, the resulting supernatant was used to determine the content of thiobarbituric acid (TBA) reactive substances (TBARS) using the OXI-TEK TBARS assay kit (Enzo Life Science, Inc., Farmingdale, NY, USA), following the provided instructions. The outcomes were normalized to the total protein quantity determined using the Bradford assay.

In addition, 8-hydroxydeoxyguanosine (8-OHdG), a biomarker for detecting DNA damage, was measured in the plasma obtained from the whole blood of each mouse using the DNA damage ELISA kit (Enzo Life Sciences, Inc., Farmingdale, NY, USA), following the manufacturer’s instructions. The obtained values were normalized to the total protein content.

### 2.6. Western Blot Analysis

The brain tissues were dissected and processed to extract the cytoplasmic and nuclear proteins using the NE-PER^®^ nuclear and cytoplasmic extraction reagent (Thermo Fisher Scientific), according to the supplier’s instructions, in the presence of the cOmplete™ Protease Inhibitor Cocktail (Roche, Basel, Switzerland). The resulting cytoplasmic and nuclear extracts were collected, and the Bradford assay was used to determine their protein quantities. Subsequently, the proteins were denatured, separated via SDS-PAGE, and transferred to a polyvinylidene difluoride membrane for Western blotting. Primary antibodies used in this study were rabbit anti-HO-1 (Abcam, Danvers, MA, USA), rabbit anti-SOD-1 (Santa Cruz Biotechnology, Dallas, TX, USA), rabbit anti-Nrf2 (Abcam), mouse anti-β-actin (Santa Cruz Biotechnology), and rabbit anti-Lamin B (Santa Cruz Biotechnology). Secondary antibodies, including horseradish peroxidase-conjugated (Thermo Fisher Scientific), were used in accordance with the specifications of the primary antibody. Protein bands were allowed to react with SuperSignal West Pico Chemiluminescent substrate (Pierce, Cheshire, United Kingdom) and subsequently visualized using LAS4000 Mini (GE Healthcare Life Sciences, Little Chalfont, UK). Densitometric analysis of the digitalized blot images was performed using Image-Studio Lite version 5.2 (LICOR Biotechnology, Lincoln, NE, USA).

### 2.7. Cell Viability Assay

A mouse hippocampal neuronal HT22 cell line was obtained from Salk Institute (La Jolla, CA, USA) [34]. The cells were maintained in Dulbecco’s modified Eagle’s medium (DMEM) (Welgene, Gyeongsan, Republic of Korea) supplemented with 10% (*v*/*v*) heat-inactivated fetal bovine serum (Gibco/Thermo Fisher Scientific, Waltham, MA, USA) and 1% penicillin–streptomycin solution (HyClone, Logan, UT, USA). Cells were detached using trypsin–EDTA (Gibco/Thermo Fisher Scientific) and subcultured when they reached approximately 70% confluency.

HT22 cells were seeded in a 96-well culture plate at a density of 2 × 10^3^ cells per well. The following day, the cells were treated with test samples and incubated for 24 h. Cell viability was determined using the CCK-8 kit (D-plus, Dongin LS, Seoul, Republic of Korea) according to the manufacturer’s instructions and as previously described [35]. The percentage of surviving cells was calculated relative to untreated cells.

### 2.8. Luciferase Reporter Assay

The HT22 cells were transfected with a plasmid vector harboring the antioxidant response element (ARE), which drives the expression of the luciferase reporter gene, as previously described, and are subsequently referred to as HT22-ARE cells [36,37,38]. To examine the transcriptional activity of the ARE, HT22-ARE cells were plated in 6-well plates at a density of 3 × 10^5^ cells per well. The following day, the cells were treated with test samples for 24 h, while sulforaphane (SFN, Sigma-Aldrich) was used as the positive control. Then, the cells were harvested, lysed, and analyzed using the luciferase assay (Promega, Madison, WI, USA). The relative luminescent values were measured using the GloMax^®^ Explorer Multimode Microplate Reader (Promega) and normalized to the total protein quantity. 

### 2.9. Determination of Intracellular ROS Level

The intracellular reactive oxygen species (ROS) concentration was quantified by measuring the oxidation level of 2′,7′-dichlorofluorescein diacetate (DCF-DA, Sigma-Aldrich) [35,36]. HT22 cells were plated in a black-bottom 96-well plate (Nunc, Rochester, NY, USA) at a density of 2.5 × 10^4^ per well and treated with test samples for 24 h. Additionally, the cells were exposed to 300 µM of *tert*-butyl hydroperoxide (*t*BHP) for 1 h before terminating the sample treatment to induce ROS production. Then, the cells were treated with 10 µM of DCF-DA at 37 °C for 30 min. After removing the excess DCF-DA, the fluorescence was measured using a fluorescence microplate reader (Infinite 200, Tecan, Grodig, Austria) at excitation and emission wavelengths of 485 nm and 535 nm, respectively. The fluorescence intensity was quantified as a relative percentage compared to the untreated control.

### 2.10. Statistical Analysis 

Statistical analyses were performed using the PRISM 9.0 software (GraphPad, Boston, MA, USA). The results were presented as either the standard deviation (SD) or the standard error of the mean (SEM). The statistical difference among groups was assessed using Student’s *t*-test or one-way analysis of variance (ANOVA) followed by post hoc analysis. In cases where ANOVA revealed significant differences, significantly different means were determined by Tukey’s honestly significant difference test. A significance value of *p* < 0.05 was utilized as the threshold for statistical significance.

## 3. Results

### 3.1. PCA Treatment Improved Learning and Memory in SCO-Treated Mice

**Figure 2:**
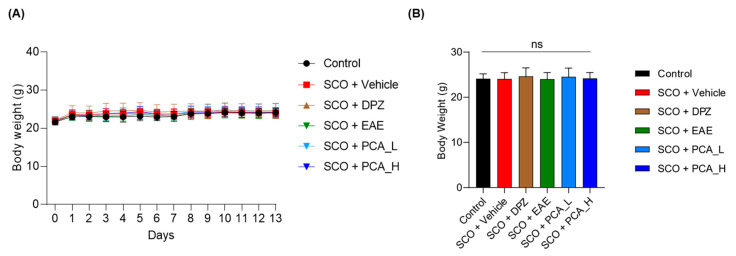
Average BWs of mice. (**A**) BWs of C57BL/6J mice regularly monitored throughout the entire experimental period. (**B**) BWs were measured at the final point before sacrifice. No significant differences were observed among the experimental groups. Data are presented as the mean ± SD (*n* = 8). Statistical analysis was performed using one-way ANOVA with Tukey’s multiple comparison tests. ‘ns’ indicates not significant at *p* < 0.05.

At 6 weeks of age, C57BL/6J mice were orally administered daily doses for 13 days (Figure 1A). The study comprised six groups based on the following treatment regimens: Control (vehicle only), SCO + vehicle (SCO alone), SCO + DPZ, SCO + EAE, SCO + PCA_L, and SCO + PCA_H (Table 1). Throughout the entire experimental period, the BW of each mouse was regularly monitored (Figure 2A). At the termination of the animal study, no significant differences were found between the average BWs among the groups (Figure 2B). Thus, the treatment for each group at the designated dose did not affect the biological condition of those experimental mice.

**Figure 3:**
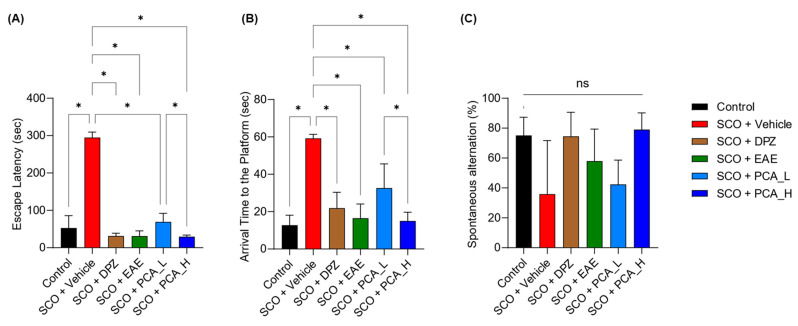
PCA treatment improved memory-related behaviors impaired in SCO-treated mice. (**A**–**C**) Behavioral assessments were conducted using the passive avoidance task (**A**), Morris water maze task (**B**), and Y-maze task (**C**). The latency for mice to escape the dark chamber where an electrical shock was given (**A**) and the arrival time of the mice to reach the platform in the pool (**B**) were significantly increased following SCO treatment. PCA or EAE administration significantly prevented these behavioral impairments. The average ratios of spontaneous alteration to navigate different paths in the Y-maze (**C**) were not significantly different among the groups. Data are presented as the mean ± SD (*n* = 8). Statistical analysis was performed using one-way ANOVA with Tukey’s multiple comparison tests. Asterisks denote statistical significance at *p* < 0.05. ‘ns’ indicates not significant.

The mice underwent behavioral assessments focusing on learning and memory capabilities (Figure 3). These assessments included the passive avoidance task to evaluate associative memory through contextual fear conditioning (Figure 3A), the Morris water maze task to test spatial learning and memory (Figure 3B), and the Y-maze task to assess the working memory (Figure 3C). These findings demonstrated that SCO affected the associative and spatial learning and memory of mice, whereas DPZ offset these effects. Importantly, a significant improvement in both associative and spatial learning and memory was observed in SCO-induced memory impairments following treatment with PCA in a dose-dependent manner (Figure 3A,B). Daily EAE treatments at 150 mg/kg BW were also effective, which was consistent with previously reported observations [6]. However, there was no significant effect on working memory across the experimental groups (Figure 3C).

### 3.2. PCA Treatment Ameliorated Hippocampal Injury in SCO-Treated Mice

**Figure 4:**
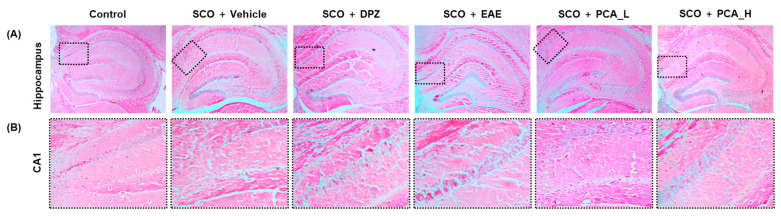
PCA treatment ameliorated histological damage in SCO-treated mice. (**A**) Representative image of the hippocampal area stained with H&E dye at 4× magnification. (**B**) Enlarged view of the CA1 region at 10× magnification (indicated by a dotted line box).

The hippocampal area is well acknowledged for its central role in learning and memory. Specifically, pyramidal neurons in the CA1 region are susceptible to both endogenous and exogenous oxidative stressors, which are closely associated with the onset of cognitive decline [39,40,41]. After the termination of behavioral assays, the mice were sacrificed. Their brains were dissected and sliced for H&E staining. Histological analysis revealed that SCO-treated mice exhibited a disrupted arrangement of neural cells in the CA1 region of the hippocampus, indicative of considerable histopathological damage. In addition, DPZ, used as a positive control, improved the structural disruption in the hippocampus. Notably, the effects of EAE at 150 mg/kg BW/day and PCA at both tested doses in ameliorating histological abnormalities in the hippocampus were comparable to those observed with DPZ (Figure 4). These findings demonstrated that PCA mitigated SCO-induced hippocampal damage.

### 3.3. PCA Treatment Decreased Oxidative Stress Levels in SCO-Treated Mice

**Figure 5:**
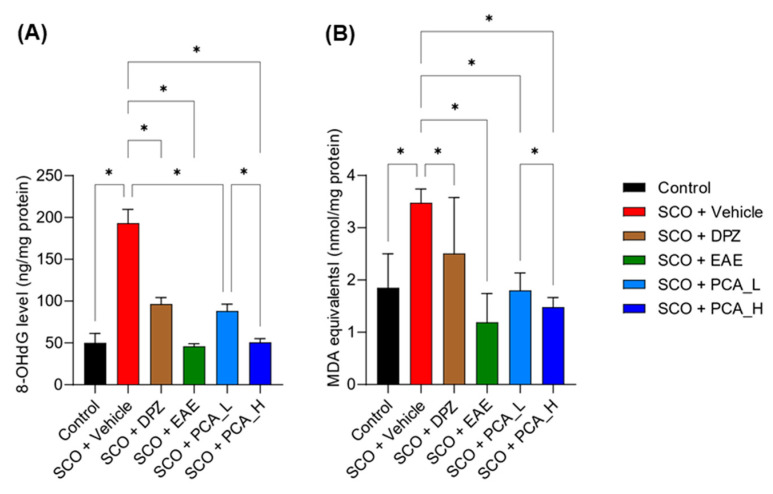
PCA treatment reduced oxidative stress in SCO-treated mice. (**A**,**B**) The levels of 8-OHdG, a marker for oxidative DNA damage, in plasma (**A**) and MDA, an end product of lipid peroxidation, in cerebral cortical tissue (**B**) were determined by ELISA. Data are presented as the mean ± SD (*n* = 5). Statistical analysis was performed using one-way ANOVA with Tukey’s multiple comparison tests. Asterisks denote statistical significance at *p* < 0.05.

SCO treatment can generate oxidative stress and synaptic dysfunction, subsequently inducing memory impairments [42,43]. To examine whether oxidative stress was decreased in SCO-treated mice after administering PCA, the 8-OHdG and MDA levels in the plasma and cerebral cortex, respectively, were measured. We found that both plasma 8-OHdG and cortical MDA levels were significantly reduced in SCO-treated mice in a dose-dependent manner following PCA administration (Figure 5). Expectedly, EAE treatment was also effective [6]. This suggested that dietary PCA reduced oxidative stress in SCO-treated mice.

### 3.4. PCA Treatment Upregulated Antioxidant Enzymes in the Hippocampus of SCO-Treated Mice

**Figure 6:**
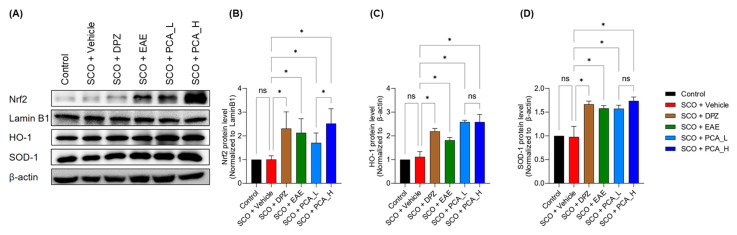
PCA treatment elevated expressions of antioxidant biomarkers in the hippocampus of SCO-treated mice. (**A**) Representative Western blot images of the hippocampal tissue homogenates. (**B**–**D**) Quantification of protein levels of nuclear Nrf2 (**B**), cytoplasmic HO-1 (**C**), and cytoplasmic SOD-1 (**D**). Data are presented as the mean ± SD (*n* = 5). Statistical analysis was performed using one-way ANOVA with Tukey’s multiple comparison tests. Asterisks denote statistical significance at *p* < 0.05. ‘ns’ indicates not significant.

Moreover, we found that Nrf2, a master regulator of the antioxidant defense mechanism, was highly activated in the hippocampus of mice exposed to PCA or EAE compared to those untreated or treated with SCO alone (Figure 6A,B and Figure S1A). Consistently, the expressions of its downstream enzymes, HO-1 (Figure 6A,C, Figures S1B and S2) and SOD-1 (Figure 6A,D and Figure S1C), were significantly increased in the hippocampus after administering PCA and EAE. These findings indicate that taking PCA directly or through PCA-containing food materials may enhance antioxidant activity, thereby mediating a defense response in the hippocampal region being challenged by oxidative stress.

### 3.5. PCA Treatment Decreased Oxidative Stress-Induced Hippocampal Neuronal Cell Death

**Figure 7:**
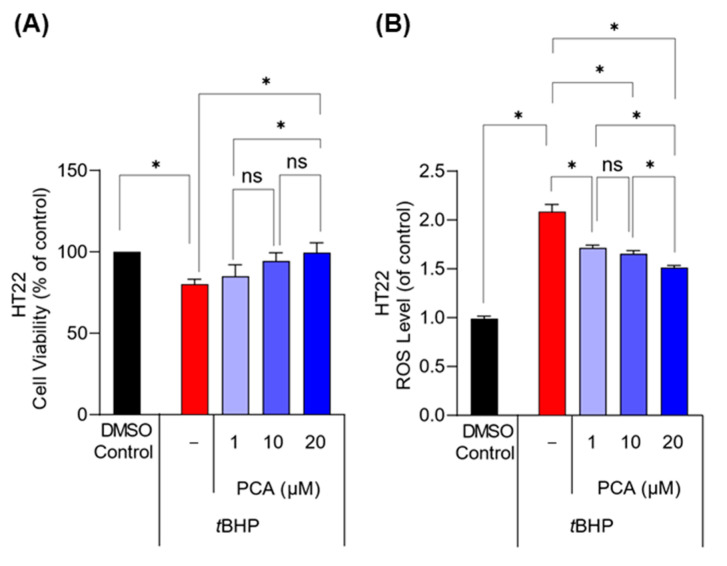
PCA treatment protected hippocampal neurons from oxidative stress-induced death. HT22 hippocampal neuronal cells were treated with PCA in the presence of *t*BHP, an ROS-producing oxidant. (**A**,**B**) *t*BHP decreased HT22 cell viability (**A**) and increased intracellular ROS levels concurrently (**B**). PCA treatment restored viability and reduced ROS levels in *t*BHP-exposed HT22 cells. Data are presented as the mean ± SEM (*n* = 3). Statistical analysis was performed using one-way ANOVA with Tukey’s multiple comparison tests. Asterisks denote statistical significance at *p* < 0.05. ‘ns’ indicates not significant.

**Figure 8:**
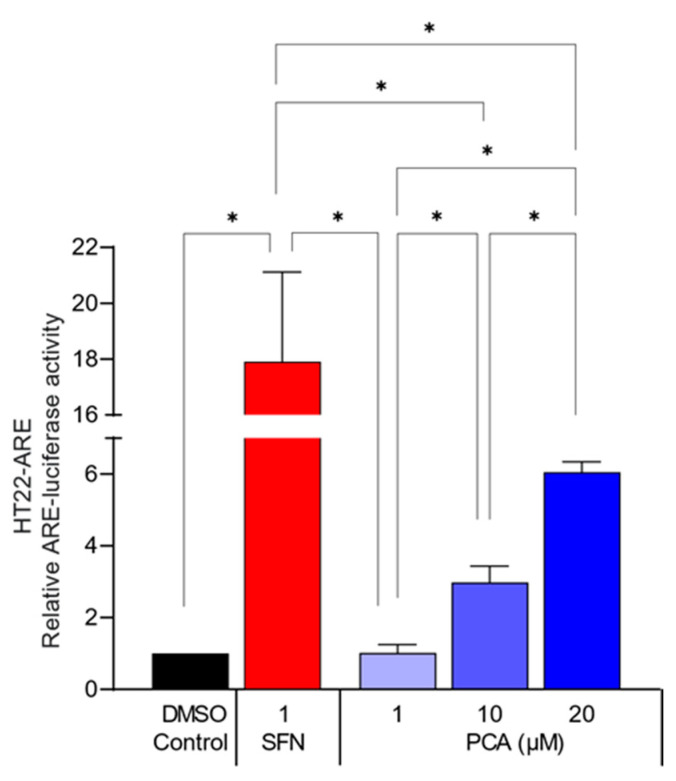
PCA treatment elevated the ARE transcription activity in hippocampal neurons. HT22-ARE cells were treated with PCA at designated concentrations. PCA treatment significantly and concentration-dependently increased the transcriptional activity of the ARE, a DNA sequence where Nrf2 binds to upregulate its downstream antioxidant genes. SFN, sulforaphane, served as a positive control. Data are presented as the mean ± SEM (*n* = 3). Statistical analysis was performed using one-way ANOVA with Tukey’s multiple comparison tests. Asterisks denote statistical significance at *p* < 0.05.

To examine whether PCA has a neuroprotective effect against oxidative stress-induced cell death, HT22 cells were exposed to an ROS producer, *t*BHP, in the absence or presence of PCA. *t*BHP at a concentration of 300 µM induced a notable decrease in cell viability accompanied by an increase in intracellular ROS levels (Figure S3A,B). Conversely, PCA at non-toxic concentrations (Figure S3C) increased cell viability and concurrently decreased the ROS levels elevated by *t*BHP (Figure 7A,B). This demonstrated that PCA protected the hippocampal neuronal cells from ROS-mediated cytotoxicity. Additionally, PCA was found to increase the ARE transcription activity in HT22-ARE cells in a concentration-dependent manner (Figure 8), which indicates an upregulation of Nrf2-mediated antioxidant defense mechanisms. 

## 4. Discussion

Oxidative stress related to aging plays a significant role in the onset and progression of neurodegenerative disorders in conjunction with protein aggregation, mitochondrial dysfunction, and inflammation [23,44,45]. The hippocampus is particularly susceptible to oxidative stress-induced damage, which is closely associated with a decline in learning and memory functions related to information encoding, consolidation, and retrieval [46,47,48]. 

Numerous research investigations have indicated that consuming phytochemicals from plant-based sources reduces the risk of developing neurodegenerative conditions [49,50]. The potential preventive impact of antioxidant activity is suggested to be a contributing factor. Various phytochemical compounds, including flavonoids, polyphenols, and carotenoids, exhibit antioxidant characteristics that aid in counteracting free radicals and diminishing oxidative stress [51,52]. 

This study evaluated the memory-improving effect of PCA, a phytochemical with antioxidant activity, using the SCO-induced acute memory impairment mouse model. SCO is well-documented for inducing oxidative stress, causing neuronal damage in the brain, and is, thus, commonly employed in research [29,30,31,32]. Our findings indicate the potential of PCA to protect against oxidative stress-induced memory deficit by upregulating the Nrf2 signaling pathway in C57BL/6J mice exposed to SCO. Particularly, oral administration of PCA at doses of 10 and 100 mg/kg BW/day and EAE at 150 mg/kg BW/day led to the mitigation of cognitive decline, potentially through the attenuation of oxidative damage.

PCA, also known as 3,4-dihydroxybenzoic acid, is a polyphenolic compound that is found in various edible fruits, vegetables, and herbs [53,54] Its presence in several medicinal plants highlights its pharmacologically beneficial characteristics. Numerous studies have demonstrated that PCA exhibits a range of beneficial effects, including antibacterial, anticancer, antihyperlipidemic, antidiabetic, anti-inflammatory, and nephroprotective properties, primarily attributed to its antioxidant capabilities [54,55,56]. Recent research indicates that PCA can reduce the expression of cleaved caspase-3, thereby inhibiting cardiomyocyte apoptosis triggered by hypoxia/reoxygenation [57]. In addition, PCA has been found to exert neuroprotective effect in PC12 cells and rat models, partly through its ability to inhibit free radical generation and activate endogenous antioxidant enzymes [58]. These findings suggest that PCA, as a dietary component, has potential benefits in mitigating oxidative stress and protecting against neural damage.

In this study, we observed that PCA protected HT22 hippocampal neuronal cells from ROS-induced oxidative stress. PCA demonstrated a concentration-dependent scavenging effect on DPPH free radicals and increased FRAP levels (Figure S4). The results obtained from CCK-8, DCF-DA, and ARE-luciferase reporter assays collectively indicated that PCA inhibited HT22 cell death induced by *t*BHP treatment by reducing intracellular ROS levels and activating the transcriptional activity of Nrf2, a key regulator of phase II antioxidant defense enzymes, including HO-1 and SOD-1 [59,60]. Nrf2 is a transcription factor responsible for controlling gene expression in antioxidant defense and detoxification mechanisms [26,61]. HO-1 is an enzyme that facilitates the breakdown of heme to produce biliverdin, carbon monoxide, and iron. Normally, Keap1 binds to Nrf2 and regulates its activity by promoting ubiquitin-mediated degradation [62]. Activating the Nrf2/HO-1 signaling pathway can safeguard neurons against oxidative stress and prevent the onset of neurodegenerative conditions [63,64]. Therefore, consistent with the aforementioned in vivo findings, PCA possesses the antioxidative potential to mitigate oxidative stress, suppress neural damage, and, thus, improve learning and memory impairments in SCO-treated mice.

In a series of studies aimed at elucidating the cognitive-improving potential of orally administered EA leaves, we discovered that EAE effectively protected hippocampal neuronal cells from oxidative stress and neuroinflammation in vivo, as tested in SCO-treated mice [6] and 5XFAD transgenic mice [7], respectively. Particularly noteworthy is the presence of PCA as an ingredient in EAE, with an average analyzed content of 20.05 ± 3.76 mg/g EAE (Figure S5). Based on this analysis, it is estimated that 150 mg of EAE contains approximately 3 mg of PCA. The administration of PCA at a dose of 10 mg/kg BW/day was significantly effective in improving learning and memory, although slightly less effective compared to EAE administration, as revealed by the behavioral, histological, and biochemical assays. 

The learning and memory improvements with EAE administration are believed to result from the combined effect of PCA and other components present in EAE, such as quercetin, rutin, and kaempferol [10,11]. These components may interact synergistically, enhancing the overall efficacy of EAE. Furthermore, the presence of these additional components may influence the bioavailability and metabolism of PCA, potentially enhancing its delivery and efficacy to hippocampal tissues. Although PCA alone also demonstrated significant neuroprotective effects, indicating its beneficial role in cognitive health interventions, the combined effects of the various constituents in EAE, including PCA, contribute to its superior effectiveness in mitigating learning and memory deficits. This suggests the value of considering whole extracts and the interactions of their components in potential treatments for cognitive decline.

PCA can be effectively maintained at relatively high concentrations in plasma through dietary intake and direct absorption in the small and large intestine without additional hydrolysis [55,65,66,67]. However, further investigation is necessary to ascertain the bioavailability of PCA and its specific role in the antioxidant potential in combination with other bioactive components in EAE. Nonetheless, this study indicates that PCA is considered safe at a dose of 100 mg/kg BW/day [54], exhibiting potent antioxidant, antibacterial, anticancer, antihyperlipidemic, antidiabetic, and anti-inflammatory properties [18,19,56,58]. Further research is imperative to evaluate its clinical reliability, safety, and efficacy.

Overall, the results of this study indicate that administering PCA can help alleviate the primary symptoms of amnesia and neural damage caused by oxidative stress. Specifically, our data emphasize that the positive effects on memory enhancement are largely due to PCA, a significant element of EAE. Furthermore, these results imply that PCA administration improves cognitive impairments by activating the Nrf2 signaling pathway.

In conclusion, our study demonstrated that oral administration of PCA at ≥10 mg/kg BW/day significantly ameliorated SCO-induced learning and memory deficits, possibly by stimulating Nrf2-mediated antioxidant and defense responses in the brain. These findings have implications for developing functional foods containing a significant quantity of PCA. Ultimately, it may enhance the food industry by ensuring compliance with quality standards, meeting regulatory requirements, and catering to the increasing consumer demand for healthier dietary options. PCA exhibits notable health advantages and may serve as a promising nutritional ingredient for mitigating neurodegenerative diseases associated with oxidative stress. 

## Figures and Tables

**Table 1:** Experimental groups of C57BL/6J mice used in this study.

Experimental Group (*n* = 8)	SCO Treatment (*i.p.*)	Sample Administration (*p.o.*)
Control (vehicle only)	–	–
SCO + vehicle (SCO alone)	+	–
SCO + DPZ	+	5 mg/kg BW/day
SCO + EAE	+	150 mg/kg BW/day
SCO + PCA_L	+	10 mg/kg BW/day
SCO + PCA_H	+	100 mg/kg BW/day

Abbreviations: *i.p.*, intraperitoneal injection; *p.o.*, per os, oral administration; DPZ, donepezil; EAE, *Euonymus alatus* leaf extract; PCA, protocatechuic acid.

## Data Availability

The original contributions presented in the study are included in the article/Supplementary Material, further inquiries can be directed to the corresponding authors.

## Supplementary Materials

The following supporting information can be downloaded at: https://www.mdpi.com/article/10.3390/foods13172664/s1, Figure S1: Whole blot images for the protein expression levels of Nrf2 (A), HO-1 (B), and SOD-1 (C), as depicted in Figure 6; Figure S2: Relative mRNA level of *ho-1* in mouse hippocampal tissue; Figure S3: Cytotoxicity tBHP and PCA in HT22 cells. (A,B) Intracellular ROS level (A) and cell viability following tBHP treatment at various concentrations (B). (C) Cell viability at different concentrations of PCA; Figure S4: Radical scavenging activity of EAE and PCA. (A) DPPH assay. (B) FRAP assay. DPPH, 2,2-diphenyl-1-picrylhydrazyl; FRAP, ferric ion reducing antioxidant power; Vit C, vitamin C; Figure S5: Representative HPLC chromatogram of EAE and its potent components.
