# Supplementary material for: Protocatechuic Acid from Euonymus alatus Mitigates Scopolamine-Induced Memory Impairment in Mice
Source: Foods. 2024 Aug 23;13(17):2664. doi: 10.3390/foods13172664 (PMC11394611; doi:10.3390/foods13172664)
Supplement: Supplementary file 1 [file foods-13-02664-s001.zip › foods-3162171-supplementary.pdf]

Supplementary information

Materials and Methods

High-performance liquid chromatography (HPLC) analysis

The dried leaves of EA were subjected to extraction using 80% v/v ethanol in sterilized water. The extraction yield was 34.2%. The freeze-dried EAE and PCA (as a standard) were dissolved in a solution comprising 0.1% formic acid in methanol to a concentration of 2 mg/mL. Then, the solutions were filtered through a 0.2 μm polyvinyl difluoride syringe filter. Analysis was conducted using an Agilent 1200 series HPLC/UV-Vis/MSD system (Santa Clara, Ca, USA) equipped with apparatuses including an auto-degasser, quaternary pump, auto-sampler, column thermostat, and photodiode array detector. The mobile phases for the analysis consisted of 0.1% trifluoroacetic acid in water (solvent A) and absolute methanol (solvent B). The HPLC analysis was performed at a wavelength of 254 nm and a flow rate of 0.7 mL/min using the Eclipse XDB-C18 column (250 mm x 4.6 mm, 5 μm). The separation protocol involved a gradient elution starting with 90–10% solvent B over 0–10 min, followed by 79–21% solvent B over 10–25 min, 0–100% solvent B over 25–35 min, and finally returning to 90–10% solvent B over 35–50 min. The PCA content in EAE (mg/g EAE) was quantified by correlating the peak area (y) with the concentration of the PCA standard.

Results

Identification of PCA in EAE using HPLC analysis

PCA exhibited an identical retention time (33.78 min) to the predominant compounds found in EAE and displayed a matching UV pattern upon scanning. Comparison with various types of phytochemicals (Suppl. Fig. S5) revealed that PCA was the principal compound in the EAE with a retention time of 33.78 min.

Supplementary Figures

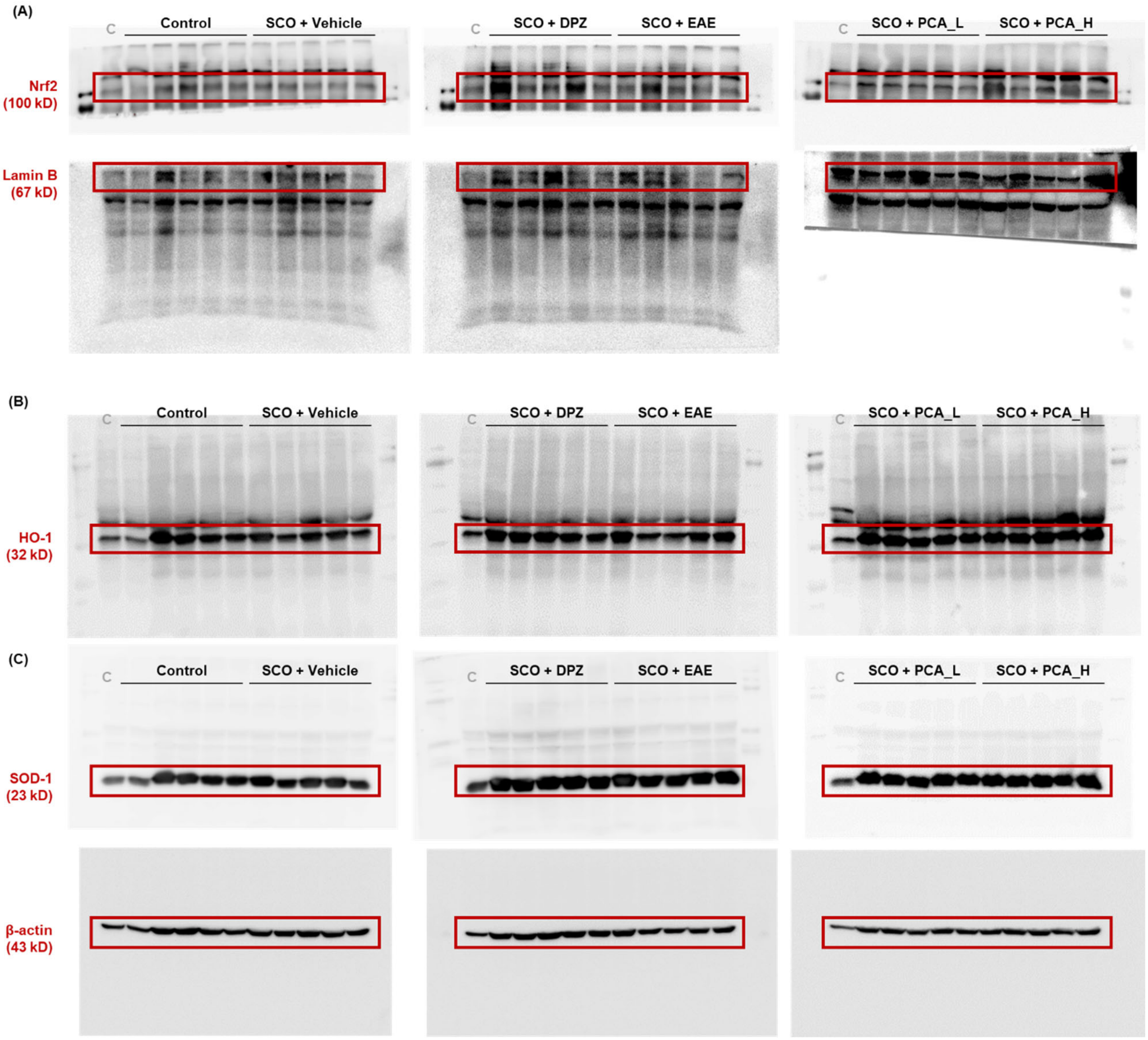

Suppl. Fig. S1. Whole blot images for the protein expression levels of Nrf2 (A), HO-1 (B), and SOD-1 (C), as depicted in Figure 6.

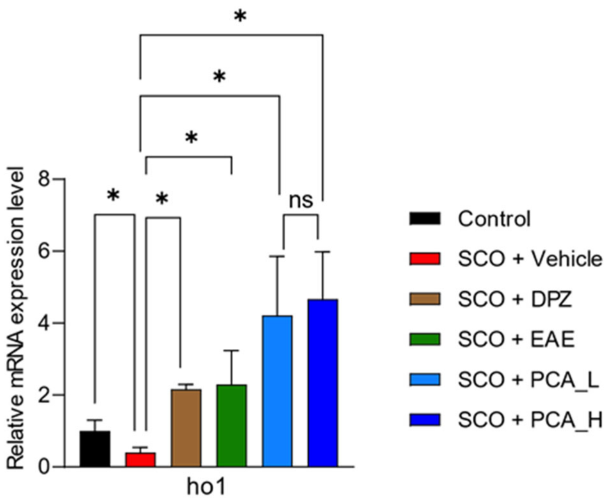

Suppl. Fig. S2. Relative mRNA level of *ho-1* in mouse hippocampal tissue.

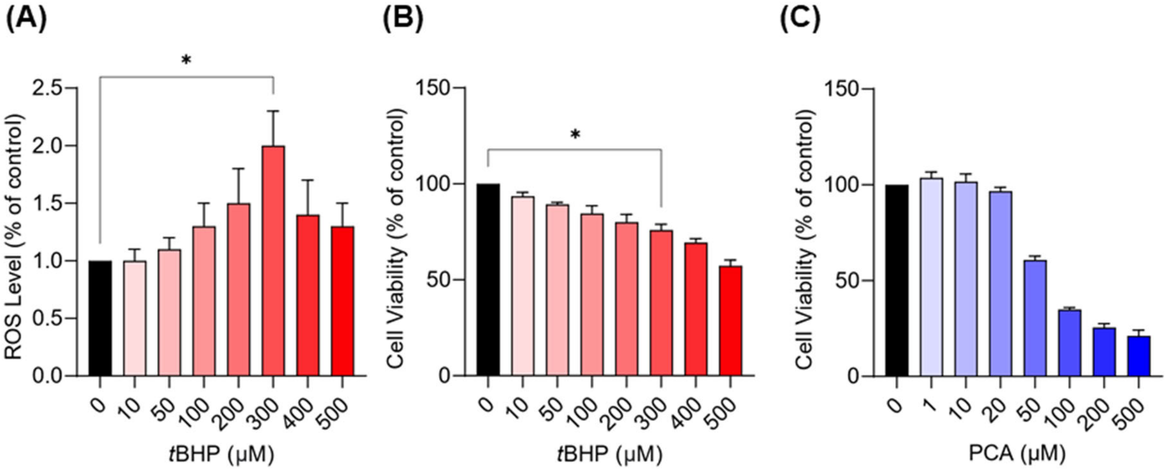

Suppl. Fig. S3. Cytotoxicity *t*BHP and PCA in HT22 cells. (A–B) Intracellular ROS level (A) and cell viability following *t*BHP treatment at various concentrations (B). (C) Cell viability at different concentrations of PCA.

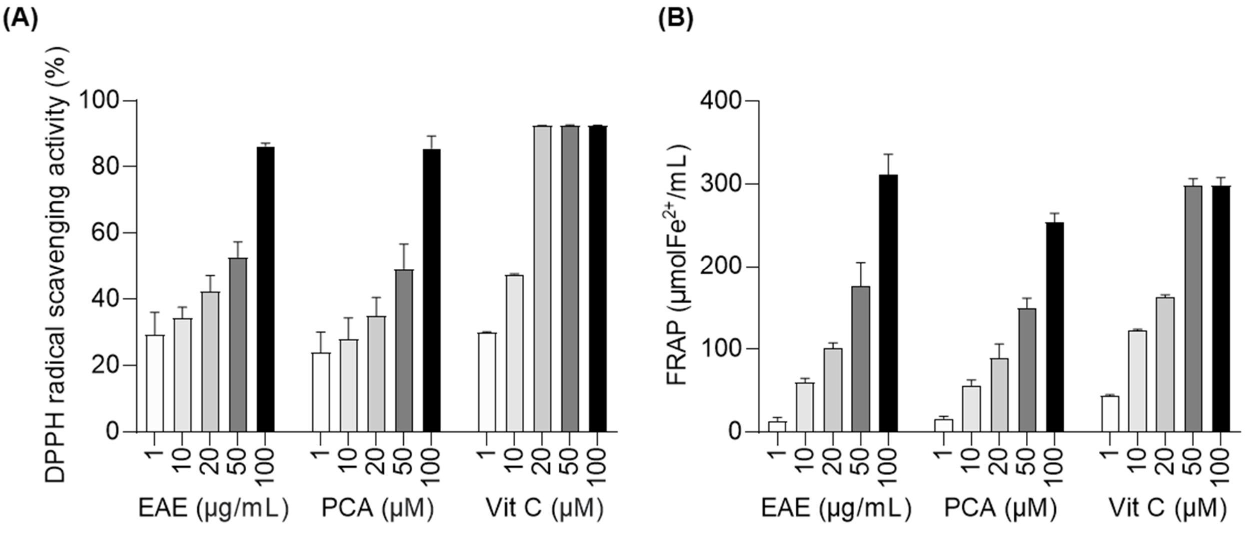

Suppl. Fig. S4. Radical scavenging activity of EAE and PCA. (A) DPPH assay. (B) FRAP assay. DPPH, 2,2-diphenyl-1-picrylhydrazyl; FRAP, ferric ion reducing antioxidant power; Vit C, vitamin C.

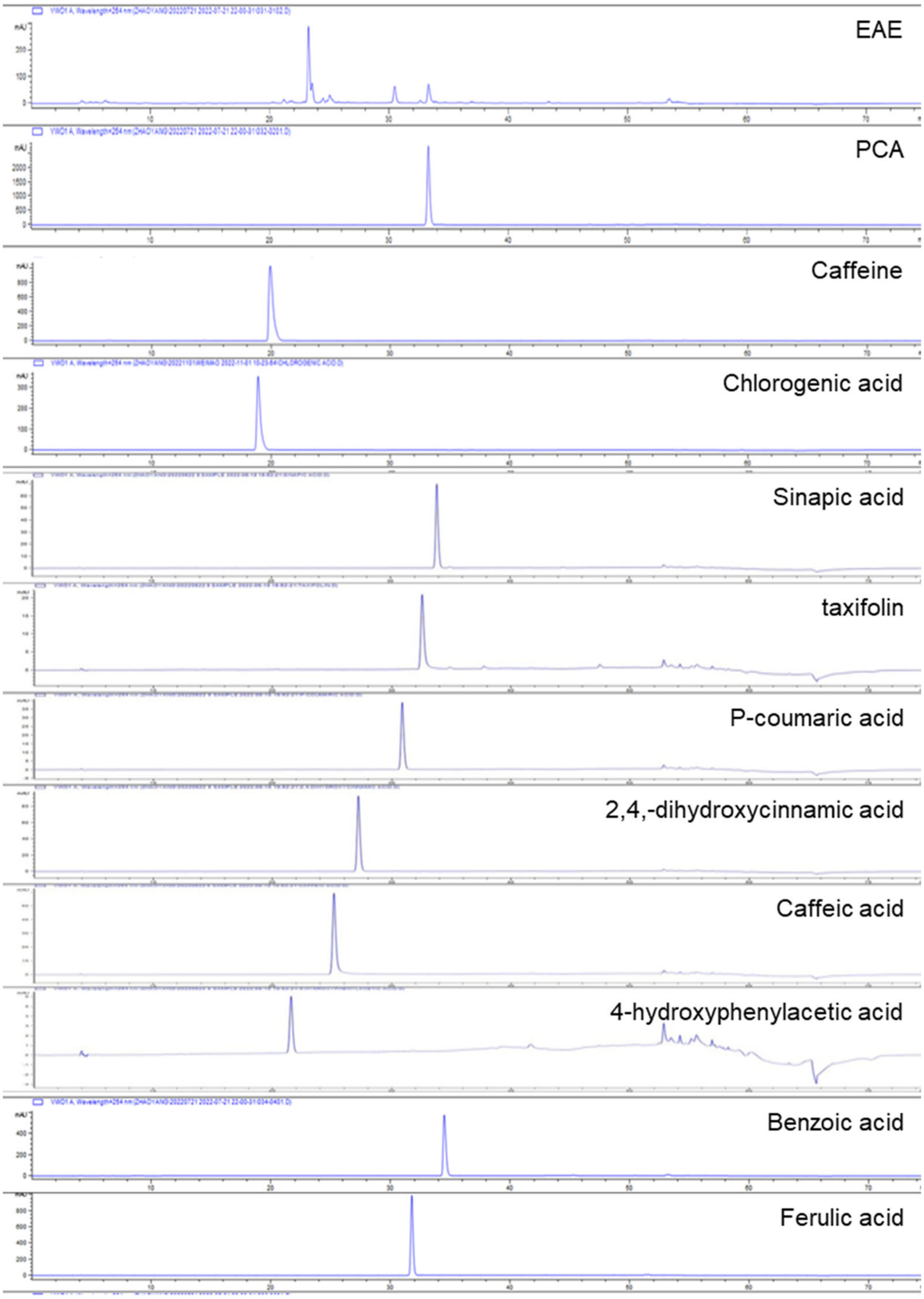

Suppl. Fig. S5. Representative HPLC chromatogram of EAE and its potent components.
